# From East to West: Effects of Respite Care Models for Long-Term Care

**DOI:** 10.1093/geroni/igaf122.849

**Published:** 2025-12-31

**Authors:** Hong Mi, Fei Sun, Joseph Gaugler

## Abstract

As populations around the world grow older, respite care has become an essential component of long-term care systems, providing temporary relief for family caregivers while supporting the well-being of individuals with care needs. This symposium examines respite care models in Eastern and Western contexts, exploring their effectiveness, policy frameworks, and cultural adaptations. By bringing together scholars, policymakers, and industry experts, the discussions aim to advance both research and practice in long-term care. A key focus will be on the health outcomes of respite care, including its effects on physical and mental well-being for both caregivers and those receiving care. Cross-cultural differences in intervention design and evaluation methods will also be explored. Another area of discussion will address the factors influencing respite care utilization, including economic conditions, policy support, and technological innovations, while identifying barriers to access and service delivery. Comparative analyses of respite care models will highlight how different long-term care insurance systems shape service provision and how cultural values influence program design and usage. Policy discussions will examine the effectiveness of government-led initiatives, incentive structures, and international best practices. Finally, the symposium will explore the role of social enterprises, digital platforms, and innovative business models in expanding and improving respite care services. By fostering interdisciplinary dialogue, this symposium aims to inform policy development and promote sustainable, person-centered respite care solutions worldwide.

